# The Risk Contagion between Chinese and Mature Stock Markets: Evidence from a Markov-Switching Mixed-Clayton Copula Model

**DOI:** 10.3390/e25040619

**Published:** 2023-04-06

**Authors:** Hongli Niu, Kunliang Xu, Mengyuan Xiong

**Affiliations:** 1 School of Economics and Management, University of Science and Technology Beijing, Beijing 100083, China; 2 School of Economics and Management, Hanjiang Normal University, Shiyan 442000, China

**Keywords:** risk contagion, Chinese stock market, mature stock markets, Markov-switching, Clayton copula

## Abstract

Exploring the risk spillover between Chinese and mature stock markets is a promising topic. In this study, we propose a Markov-switching mixed-Clayton (Ms-M-Clayton) copula model that combines a state transition mechanism with a weighted mixed-Clayton copula. It is applied to investigate the dynamic risk dependence between Chinese and mature stock markets in the Americas, Europe, and Asia–Oceania regions. Additionally, the conditional value at risk (CoVaR) is applied to analyze the risk spillovers between these markets. The empirical results demonstrate that there is mainly a time-varying but stable positive risk dependence structure between Chinese and mature stock markets, where the upside and downside risk correlations are asymmetric. Moreover, the risk contagion primarily spills over from mature stock markets to the Chinese stock market, and the downside effect is stronger. Finally, the risk contagion from Asia–Oceania to China is weaker than that from Europe and the Americas. The study provides insights into the risk association between emerging markets, represented by China, and mature stock markets in major regions. It is significant for investors and risk managers, enabling them to avoid investment risks and prevent risk contagion.

## 1. Introduction

As global stock markets become increasingly interconnected, the risk of contagion is becoming more prominent [1,2]. Measuring this contagion effectively is crucial for China and other emerging economies, which may be more vulnerable to international risk contagion, to improve their risk supervision [3,4]. Numerous scholars have studied the risk contagion between markets [5,6,7]. With the rapid development of the Chinese stock market, the largest emerging market in the world, there is growing interest in investigating the risk contagion between it and more mature markets, and several models have been developed for empirical analysis [8,9]. Traditional models have limitations in depicting the dynamic and asymmetric structures and are constrained by their ability to only show linear correlations. Consequently, scholars have turned to copula-based models to enrich research in this field, and the advantages of copula-based models over traditional models have been confirmed [10,11,12,13].

The motivation behind this work is twofold. Firstly, most existing copula-based models that evaluate risk contagion tend to focus on measuring individual tail correlations or positive dependence, which limits the analysis of the contagion mechanism from a comprehensive perspective [14,15]. While it is important to examine positive dependence, which occurs when two stock markets rise or fall in tandem, it is also crucial to consider negative dependence structures, where one market rises while the other falls, which may offer opportunities for hedging investment risks or realizing arbitrage. Overemphasizing risk contagion under one dimension may lead to a distorted perception of international markets. Therefore, it is necessary to develop a tool that provides a more comprehensive assessment of the dependence structure between markets. Secondly, the Chinese stock market has a unique profile with its late start, rapid development, and high volatility, and most existing conclusions and guidelines drawn from mature markets may not provide reliable references for its development. As emerging countries are often in a passive position in international risk contagion, investigating the risk contagion mechanism between the Chinese market and different mature markets has important reference significance [16,17,18].

Thus, to comprehensively investigate the risk contagion between Chinese and mature markets in three representative regions on a global scale (Asia–Oceania, Europe, and the Americas [19]), we attempt to construct a novel Markov-switching mixed-Clayton (Ms-M-Clayton) copula model. This model considers four types of tail correlations simultaneously and calculates the conditional value-at-risk (CoVaR) in different routes [20,21]. 

We start with a Clayton copula model that describes the upside correlation of two random variables under a positive dependence structure. We first rotate it by 90°, 180°, and 270° and weight the four individual models as a mixed-Clayton (M-Clayton) copula. The M-Clayton model can capture both upside and downside correlations between two markets under both positive and negative dependence structures. Then, to capture correlations in a time-varying manner, we introduce a two-state switching mechanism following the Markov chain. Using the estimated results of the Ms-M-Clayton copula model, we calculate the CoVaRs under the four dimensions to measure the markets’ risk spillover. The empirical results indicate that there is dynamic and generally stable positive dependence between the Chinese and mature markets, with the downside risk correlation being stronger than the upside correlation in most cases. Additionally, the risk contagion is primarily manifested in a spillover from mature markets to the Chinese market. Furthermore, the risk spillover from Asia-Oceania to China is weaker than that from Europe and the Americas, implying that Japanese and Australian markets may be potential choices for Chinese market investors to diversify investment risks. Overall, this study reveals the risk contagion effects between emerging markets, represented by China, and major mature markets. Our findings have practical and policy implications for investors and supervisors to mitigate the adverse effects of risk contagion.

This study makes several contributions to the literature on risk contagion between Chinese and mature markets. Firstly, a novel Ms-M-Clayton copula model is formulated and applied to dynamically measure the asymmetrical dependence structure between Chinese and mature markets in three global risk regions, providing a more comprehensive perspective on the risk contagion patterns between economies. Secondly, by calculating the CoVaR in four relevant scenarios based on the results of the Ms-M-Clayton copula, we quantify and compare the risk dependence and contagion between Chinese and different mature markets, respectively. Thirdly, we provide detailed explanations for the time-varying risk dependence structure and contagion. Based on the empirical results, we provide targeted insights for both emerging and mature economies on how they can defuse risk contagion and stay safe by monitoring objects with high-risk dependence.

The remainder of this paper is arranged as follows: Section 2 sorts out the existing research on the risk contagion and the related measurement methods. Section 3 introduces the construction of marginal distribution model and Ms-M-Clayton copula model. Section 4 summarizes the datasets. Section 5 reports the empirical experiments and results. Finally, this work is concluded in Section 6.

## 2. Literature Review

Despite numerous studies exploring risk contagion, the definition is still controversial [22,23]. It is commonly believed that the risk contagion is driven by heterogeneous factors such as the investors’ behaviors and expectations [24], the information bias [25], the market supervision [26], and the completeness of financial system [27].

As one of the most representative emerging economies, the Chinese market is gradually becoming international, especially since its accession to the WTO. Thus, based on different but not mutually independent definitions of risk contagion, plenty of scholars have discussed the risk contagion between Chinese and various markets by using the traditional econometric methods, such as the granger causality, generalized autoregressive conditional heteroskedasticity (GARCH), vector autoregressive model, etc. [28,29,30], but most of the methods fail to depict nonlinear dependence and capture the asymmetric relationships dynamically. Moreover, they have poor ability to measure the tail correlation reflecting the extreme risk contagion.

To overcome these shortcomings, various copula-based methods [31] are proposed to capture the dynamic and asymmetric dependencies between series. Chang [32] constructed a mixed copula of Gumbel and Clayton copula to investigate the asymmetry between the upside and downside risk correlations of crude oil spot and futures. Huang et al. [33] proposed the rotated Gumbel and Clayton copulas, which provide a flexible perspective to measure the asymmetric risk correlation. Hussain and Li [34] found that the Chinese market has stronger dependence with Asia and Europe than the US by employing stochastic copulas. Luo et al. [35] measured the multiscale financial risk association among nine stock markets by introducing empirical mode decomposition into copulas, revealing that the high-frequency fluctuation is the major contributor of contagion. Although scholars have extended copula models on the measurement of asymmetric tail correlations, most of them are still time-invariant and only suitable for depicting static relationships.

More recently, time-varying mechanisms, such as parameter autocorrelation equations and state transition probabilities, are introduced to the invariant copulas, allowing dynamic and periodic dependence analyses [32,36,37,38]. Huang et al. [39] verified the superiority of the time-varying parameter (TVP) copulas compared to traditional methods in constructing the minimum-risk portfolios from G7 countries’ markets. Chang [32] documented the non-fixed dependence between inflation rate and REIT return by constructing a Markov-switching GRG copula, while Wang et al. [40] highlighted that the negative dependence reflects the reversal effect, which is crucial to revisit the dependence structure between markets. Thus, they constructed a dependence-switching copula based on multiple Clayton copulas to examine the risk relevance between stock and foreign exchange markets. Ji et al. [41] identified the conditional dependence between energy and agricultural commodity markets and confirmed the significance of negative dependence. However, on one hand, most of the dynamic dependence-switching copulas methods are still limited to capture the positive dependence; on the other hand, the literature utilizing TVP copulas to investigate the risk contagion between Chinese and mature markets remains to be enriched.

Several studies further quantify the degree of directional risk contagion by calculating VaR and CoVaR based on the risk association captured by copula-based models, proving the function of copula-CoVaR paradigm in measuring risk contagion. Reboredo and Ugolini [42] used the CoVaR-copula method to investigate the systemic risk contagion level in European sovereign debt markets as well as the asymmetric downside and upside spillover between precious metals [43]. Xiao [44] developed a MSGARCH-EVT-copula model and computed the CoVaR to investigate the risk spillovers of Chinese market to major East Asian markets, reporting that the downside and upside spillovers are generally different between the turbulent and calm periods. Jiang et al. [19] constructed a vine-copula-GARCH-MIDAS model and computed the CoVaR to estimate the risk spillovers among multiple stock markets. Sun et al. [45] verified that the GARCH-Copula-CoVaR method is suitable for evaluating the risk contagion of international commodity markets. Therefore, it is essential to assess the risk spillovers in different routes, which can help to understand the risk contagion mechanism between markets.

In summary, although copula models provide a more flexible perspective for depicting the non-linear risk dependence between markets, most of them focus on single tail correlation or in the positive dependence structure. Positive and negative risk dependency structures provide novel insights into financial risk contagion [40,41]. In particular, tail correlations in negative dependency structures are helpful to identify risk-hedging opportunities, so it is essential to enrich research in this field. As one of the most representative emerging markets that are vulnerable in global risk contagion, the risk contagion between Chinese and global mature markets is still controversial [44]. Therefore, we formulate a Ms-M-Clayton copula model and compute the CoVaR to analyze the risk spillovers, which not only enriches the application of dependence-switching copula models but also helps to revisit the risk contagion between Chinese and mature markets around the world.

## 3. Methodology

### 3.1. Marginal Distribution Modeling

Prior to the Copula modeling that is used to capture risk dependence between markets, a marginal distribution modeling is necessary to be applied to the original financial return time series, i.e., extracting the components that can be described by econometric models and treating the residuals as risks that cannot be depicted by models. Then, the residuals are used as the input of copula model to describe the risk dependence between markets. Considering the autocorrelation, volatility clustering and leptokurtosis of financial return series, the AR-GARCH is one of the most commonly used models to describe the financial time series [46]. Moreover, compared with the normal distribution, the generalized error distribution (GED) fits the financial time series better as it captures the thick-tailed properties well. Therefore, the AR(m)-GARCH(p,q) model with GED process is employed for marginal distribution modeling, which is written as:

(1) $$\left\{\begin{array}{l} r_{t}=\phi_{0}+\sum_{i=1}^{m}\phi_{i}r_{t-i}+\varepsilon_{t}\\ \varepsilon_{t}={\sigma_{t}e}_{t},\varepsilon_{t}|I_{t-1}\sim GED\left(0,\sigma_{t}^{2},v\right)\\ \sigma_{t}^{2}=\omega +\sum_{h=1}^{p}\alpha_{h}e_{t-h}^{2}+\sum_{k=1}^{q}\beta_{k}\sigma_{t-k}^{2} \end{array}\right.$$

where $\phi_{0}$ is the unconditional mean of the return series, and $\phi_{i}$ is and autoregressive parameter, *m* denotes the lag order, and error item $\varepsilon_{t}$ follows the GED process with freedom v and conditional variance $\sigma_{t}^{2}$. $\sigma_{t}^{2}$ is expressed by the GARCH model, in which $e_{t-h}^{2}$ denotes the ARCH component, and $\sigma_{t-k}^{2}$ denotes the GARCH component. The following restrictions: (1) $\omega >0,\alpha_{h}\geq 0,\beta_{h}\geq 0$ and (2) $\sum_{h=1}^{p}\alpha_{h}+\sum_{k=1}^{q}\beta_{k}<1$ need to be met to ensure a stationary GARCH process. Following the GED, the conditional probability density function of $\varepsilon_{t}$ is given as:

(2) $$f\left(x,v\right)=\frac{ve^{-\frac{1}{2}{\left|\frac{x}{\lambda }\right|}^{v}}}{2^{-\frac{2}{v}}\lambda \Gamma \left(\frac{1}{v}\right)}$$

in which λ is the tail-thickness parameter defined as:

(3) $$\lambda ={\left[2^{-\frac{2}{v}}\Gamma \left(\frac{1}{v}\right)\Gamma \left(\frac{3}{v}\right)\right]}^{\frac{1}{2}}$$

where $\Gamma \left(\cdot \right)$ is the Gamma function. The freedom parameter v>2 when GED follows a thick-tailed distribution; the v>2 when GED follows a thin-tailed distribution; and v=2 when GED follows a normal distribution. In general, the volatility clustering in financial returns series can be effectively described by the GARCH family models with the lag order of 1 [47].

### 3.2. Markov-Switching Mixed-Clayton Copula Function

The copula model is a connecting function for multivariate marginal distributions defined in ${\left[0,1\right]}^{n}$. For example, a bivariate joint distribution function with the marginal distributions of $F_{X}\left(x\right)$ and $F_{Y}\left(y\right)$ can be defined as:

(4) $$F_{XY}\left(x,y\right)=C\left(F_{X}\left(x\right),F_{Y}\left(y\right)\right)$$

If the marginal distributions $F_{X}(x)$ and $F_{Y}\left(y\right)$ are continuous and their joint distribution function is given, the corresponding copula model $C\left(u,v\right)$ with $u=F_{X}(x)$ and $v=F_{Y}(y)$ is uniquely determined as:

(5) $$C(u,v)=H\left(F^{-1}\left(u\right),F^{-1}\left(v\right)\right)$$

Moreover, the joint density function can be obtained by

(6) $$f_{XY}\left(x,y\right)=c\left(u,v\right)f_{X}\left(x\right)f_{Y}\left(y\right)$$

where $c\left(u,v\right)=\frac{\partial^{2}C(u,v)}{\partial u\partial v}$ is the copula density function, and $f_{X}\left(x\right)$ and $f_{Y}\left(y\right)$ are the marginal densities of variables x and y. Therefore, a distribution function with N variables is composed of N univariate marginal distributions and a copula function capturing the dependence structure between the distributions.

The copula theory and method provide a flexible perspective to measure the tail dependence. To further analyze the asymmetric risk correlations, we build a mixed-Clayton (M-Clayton) copula by combining four basic Clayton copulas with 0°, 90°, 180°, and 270° rotation, respectively, under non-fixed weights. Among the rotated Clayton copulas, the Clayton copula and 180° rotated Clayton copula are used to measure the positive dependence reflected by the lower–lower tail and higher–higher tail correlation, while the 90° and 270° rotated Clayton copulas are used to measure the negative dependence reflected by the lower–upper tail and upper–lower tail correlation. The two copulas are defined as:

(7) $$C_{1}\left(u,v,\alpha_{1},\alpha_{3}\right)=0.5C_{c0}\left(u,v;\alpha_{1}\right)+0.5C_{c180}\left(u,v;\alpha_{3}\right)$$

(8) $$C_{2}\left(u,v;\alpha_{2},\alpha_{4}\right)=0.5C_{c90}\left(u,v;\alpha_{2}\right)+0.5C_{c270}\left(u,v;\alpha_{4}\right)$$

where

(9) $$\left\{\begin{array}{l} C_{c0}\left(u,v;\alpha_{1}\right)={\left(u^{-\alpha_{1}}+v^{-\alpha_{1}}-1\right)}^{-\frac{1}{\alpha_{1}}}\\ C_{c90}\left(u,v;\alpha_{2}\right)=u-{\left[u^{-\alpha_{2}}+{\left(1-v\right)}^{-\alpha_{2}}-1\right]}^{-\frac{1}{\alpha_{2}}}\\ C_{c180}\left(u,v;\alpha_{3}\right)=u+v-1+{\left[{\left(1-u\right)}^{-\alpha_{3}}+{\left(1-v\right)}^{-\alpha_{3}}-1\right]}^{-\frac{1}{\alpha_{3}}}\\ C_{c270}\left(u,v;\alpha_{4}\right)=v-{\left[{\left(1-u\right)}^{-\alpha_{4}}+v^{-\alpha_{4}}-1\right]}^{-\frac{1}{\alpha_{4}}} \end{array}\right.$$

Thus, a M-Clayton copula can be obtained by weighting $C_{1}$ and $C_{2}$ copulas as:

(10) $$C_{M}\left(u,v,\theta \right)=\omega C_{1}\left(u,v;\alpha_{1},\alpha_{3}\right)+\left(1-\omega \right)C_{2}\left(u,v;\alpha_{2},\alpha_{4}\right)$$

where the $\theta =\left(\alpha_{1},\alpha_{2},\alpha_{3},\alpha_{4}\right)\in \left(0,+\infty \right)$, denoting the parameters of the four separate copulas, the greater the $\alpha_{1},\alpha_{2},\alpha_{3},\alpha_{4}$, the stronger the correlation. ω∈[0,1] is used to determine the weights of the $C_{1}$ and $C_{2}$.

Affected by time-varying fundamental information, the correlation and possible structural changes between financial markets is usually not static. Therefore, a state-switching mechanism assumed to be subject to Markov chain is introduced to further capture the dynamic correlation and potential dependence structural changes. We assume that there are two dependence states between financial markets [48], and the Ms-M-Clayton copula can be expressed as:

(11) $$C_{Ms-M}\left(u,v;\theta^{S_{t}}\right)=\omega^{S_{t}}C_{1}\left(u,v;\alpha_{1}^{S_{t}},\alpha_{3}^{S_{t}}\right)+\left(1-\omega^{S_{t}}\right)C_{2}\left(u,v;\alpha_{2}^{S_{t}},\alpha_{4}^{S_{t}}\right)$$

where $S_{t}$ denotes the state variable and is assumed as the following Markov transition probability [48]:

(12) $$\left\{\begin{array}{c} P_{11}=P\left(S_{t}=1|S_{t-1}=1\right)=\frac{exp(\pi_{1})}{1+exp(\pi_{1})}\\ P_{12}=P\left(S_{t}=2|S_{t-1}=1\right)=\frac{1}{1+exp(\pi_{1})}\\ P_{21}=P\left(S_{t}=1|S_{t-1}=2\right)=\frac{1}{1+exp(\pi_{2})}\\ P_{22}=P\left(S_{t}=2|S_{t-1}=2\right)=\frac{exp(\pi_{2})}{1+exp(\pi_{2})} \end{array}\right.$$

### 3.3. Markov-Switching Mixed-Clayton Copula Function

In the Ms-M-Clayton copula, the correlations of lower–lower tail, lower–higher tail, higher–higher tail, and higher–lower tail are provided as follows [49]:

(13) $$\left\{\begin{array}{l} \lambda_{MS-M}^{LL}=\underset{\alpha \to 0}{\lim}P\left(V\leq \alpha |U\leq \alpha \right)=0.5\omega^{s_{t}}2^{-\frac{1}{\alpha_{1}}}\\ \lambda_{MS-M}^{LU}=\underset{\alpha \to 0}{\lim}P\left(V\geq 1-\alpha |U\leq \alpha \right)=0.5\left(1-\omega^{s_{t}}\right)2^{-\frac{1}{\alpha_{2}}}\\ \lambda_{MS-M}^{UU}=\underset{\alpha \to 1}{\lim}P\left(V\geq \alpha |U\geq \alpha \right)=0.5\omega^{s_{t}}2^{-\frac{1}{\alpha_{3}}}\\ \lambda_{MS-M}^{UL}=\underset{\alpha \to 1}{\lim}P\left(V\leq 1-\alpha |U\geq \alpha \right)=0.5\left(1-\omega^{s_{t}}\right)2^{-\frac{1}{\alpha_{4}}} \end{array}\right.$$

### 3.4. Parameter Estimation Method

We employ the maximum-likelihood (ML) function [50] as the basis for estimating parameters. Given that there are 12 parameters to be estimated, and a traditional approach, such as the interior-point method, easily falls into local optimum, we apply the genetic algorithm (GA) that performs well in global optimization of high-dimensional parameters to exact the solution of the model [51].

Referring to Equation (6), the joint probability density function of the Ms-M-Clayton copula model with variables x and y is given as:

(14) $$f_{XY}\left(x,y\right)=\sum_{S_{t}=1}^{2}f_{X}\left(x\right)f_{Y}\left(y\right)c\left(u,v,\theta^{S_{t}}\right)P\left(S_{t}\right)$$

where $P\left(S_{t}\right)$ is the prediction probability of $S_{t}$ at time t−1. $P\left(S_{t}=1\right)$ and $P\left(S_{t}=2\right)$ are defined as [52]:

(15) $$P\left(S_{t}=1\right)=P_{11}*\left[\frac{c_{t-1}^{1}P\left(S_{t-1}=1\right)}{c_{t-1}^{1}P\left(S_{t-1}=1\right)+c_{t-1}^{2}P\left(S_{t-1}=2\right)}\right]+P_{21}*\left[\frac{c_{t-1}^{2}P\left(S_{t-1}=2\right)}{c_{t-1}^{1}P\left(S_{t-1}=1\right)+c_{t-1}^{2}P\left(S_{t-1}=2\right)}\right]$$

(16) $$P\left(S_{t}=2\right)=1-P\left(S_{t}=1\right)$$

where $c_{t-1}^{1}$ and $c_{t-1}^{2}$ represent the conditional probability density functions of the copula function in state 1 and state 2, respectively, at time t−1. Then the logarithmic likelihood function of the copula model is expressed as:

(17) $$lnL=\sum_{t=1}^{T}lnc\left(u,v;\theta^{S_{t}}\right)P\left(S_{t}\right)+\sum_{t=1}^{T}lnf_{X}\left(x\right)+\sum_{t=1}^{T}lnf_{Y}\left(y\right)$$

### 3.5. VaR and CoVaR

This work employs the value-at-risk (VaR) to measure the downside and upside risks, which indicates the maximum loss that an investor may suffer within a certain time horizon and significant level by holding a long or a short position. For return series $r_{t}$, we calculate the VaR based on its marginal distribution. With a given tail probability α, the ${VaR}_{D}^{\alpha ,t}$ and ${VaR}_{U}^{\alpha ,t}$ at time *t* is calculated by $P\left(r_{t}\leq {VaR}_{D}^{\alpha ,t}\right)=\alpha$ and $P\left(r_{t}\geq {VaR}_{U}^{\alpha ,t}\right)=1-\alpha$ respectively, which is formulated as:

(18) $$\left\{\begin{array}{l} {VaR}_{D}^{\alpha ,t}=\mu_{t}+\sigma_{t}\cdot F_{v}^{-1}\left(\alpha \right)\\ {VaR}_{U}^{\alpha ,t}=\mu_{t}+\sigma_{t}\cdot F_{v}^{-1}\left(1-\alpha \right) \end{array}\right.$$

where $\mu_{t}$ and $\sigma_{t}$ represent the conditional mean and standard deviation determined by the marginal distribution model, and $F_{v}^{-1}\left(\alpha \right)$ is the α-quantile of GED.

The conditional VaR (CoVaR) is used to capture the risk spillover between markets [42]. The CoVaR is calculated based on the measurement of copula model, reflecting the VaR of a market conditional on the extreme volatility in another market. Let $r_{t}^{i}$ and $r_{t}^{j}$ denote the return series of market i and j, and the CoVaR in four different market statuses can be expressed as follows:

(19) $$\left\{\begin{array}{c} P\left(r_{t}^{i}\leq {CoVaR}_{\left.iD\right|jD}^{\beta ,t}\left|r_{t}^{j}\leq {VaR}_{jD}^{\alpha ,t}\right.\right)=\beta \\ P\left(r_{t}^{i}\geq {CoVaR}_{\left.iU\right|jD}^{\beta ,t}\left|r_{t}^{j}\leq {VaR}_{jD}^{\alpha ,t}\right.\right)=\beta \\ P\left(r_{t}^{i}\leq {CoVaR}_{\left.iD\right|jU}^{\beta ,t}\left|r_{t}^{j}\geq {VaR}_{jU}^{\alpha ,t}\right.\right)=\beta \\ P\left(r_{t}^{i}\geq {CoVaR}_{\left.iU\right|jU}^{\beta ,t}\left|r_{t}^{j}\geq {VaR}_{jU}^{\alpha ,t}\right.\right)=\beta \end{array}\right.$$

where ${CoVaR}_{\left.iD\right|jD}^{\beta ,t}$ and ${CoVaR}_{\left.iU\right|jD}^{\beta ,t}$ represent the downside and upside VaRs of market i conditional on the extreme downside movement of market j given a confidence level β, while ${CoVaR}_{\left.iD\right|jU}^{\beta ,t}$ and ${CoVaR}_{\left.iU\right|jU}^{\beta ,t}$, respectively, represent downside and upside VaR of market i conditional on the extreme upside movement of market j given a confidence level β. 

For example, the first row in Equation (19) can be written as:

(20) $$\frac{F_{r_{t}^{i}r_{t}^{j}}({CoVaR}_{\left.iD\right|jD}^{\beta ,t},{VaR}_{jD}^{\alpha ,t})}{F_{r_{t}^{j}}({VaR}_{jD}^{\alpha ,t})}=\beta$$

Therefore, the CoVaR requires the joint distribution function of $r_{t}^{i}$ and $r_{t}^{j}$, and it can be represented by a copula function as Equation (4). Thus, Equation (19) can be written as:

(21) $$\left\{\begin{array}{l} C\left(F_{r_{t}^{i}}\left({CoVaR}_{\left.iD\right|jD}^{\beta ,t}\right),\alpha \right)=\alpha \beta \\ C\left(F_{r_{t}^{i}}\left({CoVaR}_{\left.iU\right|jD}^{\beta ,t}\right),\alpha \right)=\alpha -\alpha \beta \\ F_{r_{t}^{i}}\left({CoVaR}_{\left.iD\right|jU}^{\beta ,t}\right)-C\left(F_{r_{t}^{i}}\left({CoVaR}_{\left.iD\right|jU}^{\beta ,t}\right),1-\alpha \right)=\alpha \beta \\ F_{r_{t}^{i}}\left({CoVaR}_{\left.iU\right|jU}^{\beta ,t}\right)-C\left(F_{r_{t}^{i}}\left({CoVaR}_{\left.iU\right|jU}^{\beta ,t}\right),1-\alpha \right)=\alpha -\alpha \beta \end{array}\right.$$

Hence, the value of $F_{r_{t}^{i}}\left({CoVaR}_{\left.iD\right|jD}^{\beta ,t}\right)$ can be inferred by inverting the copula function for given values of α and β, which is denoted as ${\hat{F}}_{r_{t}^{i}}\left({CoVaR}_{\left.iD\right|jD}^{\beta ,t}\right)$, and the value of CoVaR can be inferred by inverting the marginal distribution function of $r_{t}^{i}$ as ${CoVaR}_{\left.iD\right|jD}^{\beta ,t}=F_{r_{t}^{i}}^{-1}\left({\hat{F}}_{r_{t}^{i}}\left({CoVaR}_{\left.iD\right|jD}^{\beta ,t}\right)\right)$. Similarly, the other three types of CoVaR can be obtained. To validate the significance of the risk contagion, the Kolmogorov–Smirnov (K-S) test [20] is employed to implement the significance test.

## 4. Data and Descriptive Statistics

This work adopts the China Securities Index 300 (CSI300), an important financial index jointly released by the Shanghai and Shenzhen Stock Exchanges on 8 April 2005 to represent the Chinese stock market. It consists of 300 stocks, accounting for approximately 70% of the total market capitalization of the Shanghai and Shenzhen stock markets. Compared with other stock indexes in China, the issuers of the constituent stocks in CSI300 are mostly mature companies that have the characteristics of strong resistance to manipulation, lower volatility, and strong liquidity. Therefore, it comprehensively reflects the performance of the Chinese stock market. According to [19], three risk areas, including Asia–Oceania, Europe, and the Americas, can be identified in risk contagion. Therefore, the S&P500 and GSPTSE indexes are selected to represent the Americas market, the DAX30 and FTSE100 indexes are selected to represent the European market, and the Nikkei225 and ASX200 indexes are selected to represent the Asia–Oceania market. The monthly price time series collected from Wind database are used for empirical analyses because: (1) it covers less noises than the daily and weekly prices and is widely employed in copula modeling, and (2) it contains more trend information than the yearly prices but does not suffer from manipulation [14,20,48]. The period is from July 2005, when CSI300 is officially released, to December 2020, with 186 data points containing multiple economic cycles and economic events. The logarithmic returns series $r_{t}$ reflecting the level of price changes are calculated as: $r_{t}=\left(lnP_{t}-lnP_{t-1}\right)\times 100\%$, where $P_{t}$ denotes the price at the end of month *t*.

**Figure 1:**
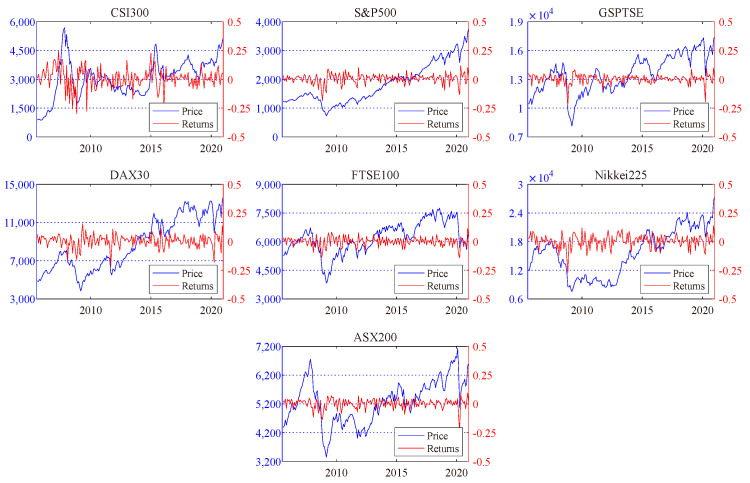
Monthly prices and returns of the selected indexes.

Figure 1 reports the prices and returns of the selected stock indexes. First, the stock market volatility in the same region is relatively similar, but those in different regions are quite different. Second, due to the global emergencies during the sample period, such as the global financial crisis, the European debt crisis, and the COVID-19 epidemic, the markets experienced several large fluctuations simultaneously, implying the potential risk contagion between Chinese and mature markets. Third, the volatility of Chinese market is significantly higher than mature markets, which may be caused by the large gap between Chinese and mature stock markets in terms of the completeness of risk supervision and the professionalism of market participants.

Table 1 reports the descriptive statistics of the return series, in which their average values are all positive. The CSI300 has the highest monthly average return with 0.0096, followed by the S&P500 and the DAX30, while the FTSE100 has the lowest monthly average return. The CSI300 has the highest volatility, with the standard deviation of 0.0858, followed by the Nikkei225. The lowest standard deviation 0.0402 is observed in the FTSE100. Moreover, the skewness statistics are all less than 0, suggesting that all the return series are featured as a long tail to the left, and there are more extreme negative returns. The skewness values of the Nikkei225 and the ASX200 are larger than others, and that of the CSI300 is closer to 0. Meanwhile, the Nikkei225 and the ASX200 have the highest kurtosis, implying the leptokurtosis feature in Asia–Oceania market is more prominent. The Jarque-Bera (J-B) test confirms that all return series are not normally distributed but featured as leptokurtosis. The Pearson correlation coefficients between CSI300 and other indexes proves a weak but positive correlation between Chinese and mature markets, and the correlations between Chinese market and the Americas, Asia–Oceania, and the European markets decreases in turn.

## 5. Empirical Results

This study uses Eviews 9 to perform a marginal distribution estimation and output the residual series and MATLAB 2018 to fit copula models.

### 5.1. Marginal Distribution Estimation

A diagnostic test on stationarity, autocorrelation, and heteroscedasticity needs to be conducted before marginal distribution modeling. The results are reported in Table A1 (seen in Appendix A), showing that all the return series are stationary by ADF, PP, and KPSS tests. According to the Ljung–Box test, only the CSI300 have autocorrelation. The Q2(P) and ARCH(P) statistics ensure the presence of ARCH effects in all series except the DAX30. Thus, AR-GARCH is suitable to fit the marginal distribution.

Considering the significance of parameters and the results of diagnostic test, the results of marginal distribution are provided in Panel A of Table A2 (see Appendix A). Most coefficients are significant at 5% level. Panel B of Table A2 reports the diagnostic results for the residuals, in which the autocorrelation and conditional heteroscedasticity are effectively overcome. Then, the standard residues are employed to conduct the risk dependence analyses with copula models.

### 5.2. Dynamic and Asymmetric Dependence Measured by MS-M-Clayton Copula

The M-Clayton copula model is first employed to measure both positive and negative dependence structures (Wang et al., 2013; Ji et al., 2018), and Table 2 reports the results, in which all parameters are significant at the 1% level. It is worth noting that the weight parameter ω across different pairwise returns is various, indicating that the existence of negative dependence between Chinese and mature stock markets. Therefore, how to recognize the occurrence of different risk dependence structures and correlations has become an urgent problem to be clarified.

Table 3 further reports the estimated results of the MS-M-Clayton copula model, where the model outperforms the invariant M-Clayton copula in terms of the logarithmic likelihood values. Most of the estimated parameters are significant at the 10% level, meaning that there are not only both positive and negative dependence structures but also dependence-switching between Chinese and mature stock markets. Overall, the risk dependence structures and correlations are different in each dependence state. Taking the CSI300-S&P500 as an example, the $P_{22}$ of 0.864 is significant and higher than $P_{11}$, meaning that state 2 is the dominant dependence structure. Similarly, for the CSI300-GSPTSE, CSI300-FTSE100, and CSI300-Nikkei225 pairs, state 2 plays a dominant role, while state 1 is dominant in CSI300-DAX30 and CSI300-ASX200 pairs.

Table 4 reports the tail correlation coefficients based on the constructed copula. Specifically, the values of $\lambda_{UU}$ are larger than that of $\lambda_{LL}$ between CSI300 and S&P500, DAX30, and Nikkei225, meaning that the upside risk correlation triggered by positive factors is stronger than the downside risk correlation triggered by negative factors, while the opposite relationship occurs between CSI300 and GSPTSE, FTSE100, and ASX200. Moreover, compared with the Americas and European mature markets, the downside risk correlation between Chinese and Asia–Oceania markets manifesting in synchronized decline is the lowest, which is usually paid special attention in practice. Although the negative dependence is not in dominant in the dominant state, it is still asymmetric. Specifically, the upper–lower tail correlation between CSI300 and S&P500, FTSE100, and Nikkei225 is stronger than the lower–upper tail correlation, indicating the probability of extreme rises in Chinese market when extreme declines occur in the three mature markets. The opposite situation can be found between CSI300 and DAX30. As for the main dependence state between CSI300 and GSPTSE and ASX200 returns, the negative dependence correlation is not observed. Therefore, during the period of smooth economic operation denoted by the main state, except for monitoring the positive risk spillover, Chinese investors and managers should pay close attention to investment opportunities in the declines of S&P500, FTSE100, and Nikkei225 while managing exposure carefully in the rises of DAX30.

**Figure 2:**
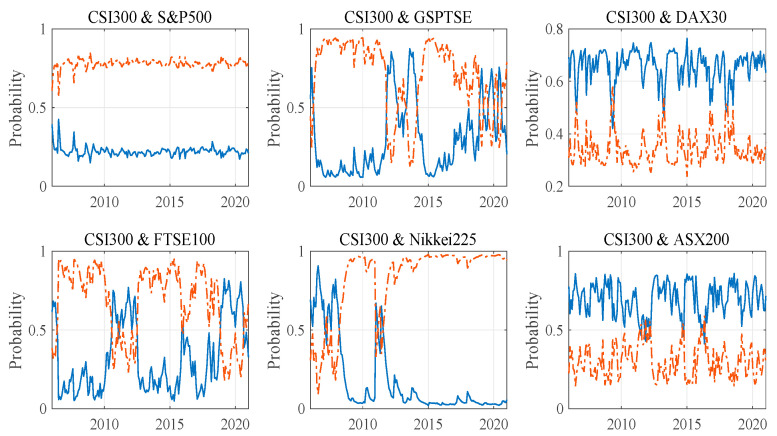
State transition probabilities between Chinese and mature markets (The blue line represents state 1, and the orange line represents state 2).

Figure 2 provides the trajectories of $P^{S_{1}}$ and $P^{S_{2}}$, in which the state transitions are observed in the risk dependence between Chinese and most mature markets. For CSI300-S&P500, there is no state-switching, and state 2 is dominant during the entire sample period, implying the stable dependence and risk correlation between the two markets. For CSI300-GSPTSE, the state transitions occur concentrated in the periods from 2013 to 2015, corresponding to cyclical financial market bubbles and the post-COVID-19 [3], in which the secondary state should be paid more attention because more investment opportunities appear with a stronger upside tail correlation and a downside tail correlation close to 0. The state transitions of CSI300-DAX30 appear periodically around 2009 (may be affected by European debt crisis) with weak persistence [53]. In the secondary state, the upside tail correlation is significant, while the downside correlation decreases to near 0, increasing the investment motivation. For CSI300-FTSE100, state 2 with apparent downside risk correlation is dominant in most of the period. However, state 1 with both upside and downside risk correlations switches to be the main dependence structure temporarily around 2009 (European debt crisis) and since the COVID-19 epidemic [53]. For CSI300-Nikkei225, state 1 with reversal correlation was the main state before 2009 and in 2012, corresponding to the global financial crisis and the Asian financial turmoil led by the exchange rate system, respectively [54]. However, state 2 with positive dependence structure plays a dominant role in most of the period, especially in recent years. For CSI300-ASX200, state 1 with a relatively low tail correlation is dominant. The state-switching process occurs around 2012 and 2015 temporarily, which is accompanied by an increase in positive risk correlation caused by regional financial turmoil [54]. Moreover, in the comparison between markets in different regions, the Asia–Oceania markets have the relatively low risk association, especially the downside risk correlation that is paid much attention in practice, with the Chinese market.

### 5.3. Comparative Analysis

#### 5.3.1. Static Dependence Measured by Invariant Copula Models

To explain the similarity and differences between our findings and previous research, we first employ seven commonly used invariant copulas, including the Gaussian, Student’s t, Gumbel, 180° rotated Gumbel, Clayton, 180° rotated Clayton, and SJC copulas [53] to measure the risk dependence between Chinese and mature markets. The estimated results of invariant copulas are reported in Table 5. 

According to the logarithmic likelihood values, it is found that the Clayton copula performs the best with significant estimated parameters, followed by the 180° rotated Gumbel copula, the Student’s t copula, and the Gaussian copula, successively, and the Gumbel copula and 180° rotated Clayton copula perform the worst. In the SJC copula measuring asymmetric positive dependence, the lower tail correlations are larger than the upper ones, but most parameters are not significant. The results suggest a positive but asymmetric risk dependence between Chinese and mature markets, and the downside correlation is stronger than the upside correlation. Overall, the results are generally consistent with the findings drawn from M-Clayton and MS-M-Clayton copulas but fail to capture the negative dependence structure and the upside correlations between CSI300 and S&P500, DAX30, and Nikkei225 effectively. Moreover, the static copulas are unable to capture the time-varying or dependence-switching characteristics of the correlations.

#### 5.3.2. Dynamic Dependence Measured by Time-Varying Parameter Copula

To assess the dynamic risk dependence correlation between Chinese and mature markets, Table 6 further reports the estimated results of four TVP copulas, in which most of the estimated parameters are significant at the 10% level. It can be found that TVP copulas perform better than the corresponding invariant copulas. Specifically, the TVP-180° rotated Gumbel copula describing the lower–lower tail correlation effectively captures the risk dependence between Chinese and mature markets, and the TVP-SJC copula also proves that the lower–lower correlation is more significant. The results confirm the positive risk dependence structure and the prominent downside risk correlation between Chinese and mature markets. The effectiveness of time-varying mechanism in depicting the dynamic risk correlation is also verified. Although TVP copulas provide an analytical view on dynamic risk correlation, a significant difference between them and the proposed MS-M-Clayton copula is that the potential negative dependence structure is not effectively depicted.

### 5.4. Asymmetric Risk Spillover Measurement by VaR, CoVaR and Nomalized CoVaR

**Figure 3:**
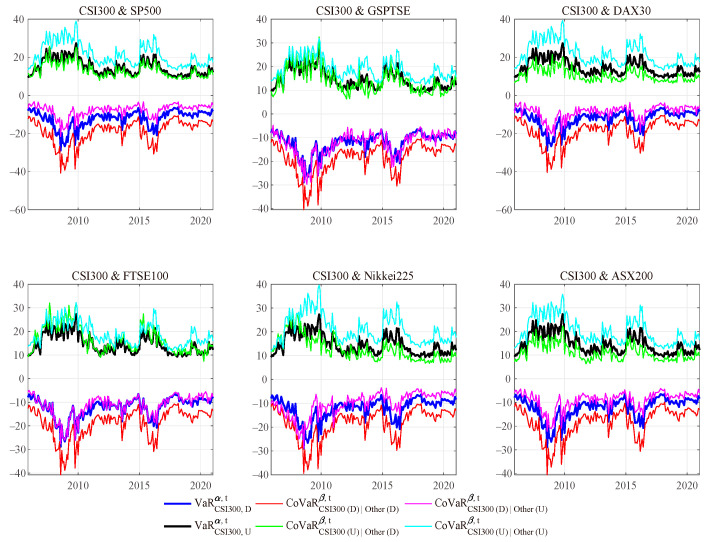
The dynamic trajectories of the VaR and the CoVaR.

To provide implications for risk supervision and portfolio risk management, we studied the extreme risk spillovers between Chinese and mature stock markets in different routes by VaR and CoVaR based on the information from marginal distribution and Ms-M-Clayton copula model. We set α and β equal to 0.05 for downside CoVaR and 0.95 for the upside CoVaR calculation. Table 7 reports the summary statistics of the VaR and the CoVaR, and Figure 3 shows the dynamic trajectories for intuitive observation.

For stock index pairs except CSI300-FTSE100, the absolute values of upside VaR and CoVaR are larger than those of the downside, respectively, meaning that the upside risk is larger than the downside risk in Chinese market. Moreover, the VaR and CoVaR show phased extreme fluctuations, which may be related to the macroeconomic uncertainties, such as the periods around 2008, 2013, and 2015. For the positive risk contagion (3 and 6 rows in Table 7), the absolute values of CoVaR are all greater than that of VaR when measuring either upside or downside risks, indicating the synergistic risk spillover from mature markets to the Chinese market. In the measurement of negative risk contagion (4–5 rows in Table 7), the absolute values of CoVaR are generally smaller than that of VaR, implying the weak existence of reverse risk spillovers. Overall, the positive risk contagion from mature markets to the Chinese market are more significant than the negative contagion. It is noteworthy that the downside risk contagion between Chinese and Asia–Oceania markets is relatively weak, suggesting that the Asia–Oceania market can be considered as a potential choice for investors in the Chinese market to diversify their investment portfolios. Table 8 further reports the hypothesis testing results by K-S test, and the statistics are generally significant at 10% level, rejecting the null hypothesis that VaR is equal to CoVaR.

To further evaluate the intensity of risk spillovers in different routes and analyze its asymmetry, Table 9 reports the summary statistics of the CoVaR normalized by VaR (CoVaR/VaR). It can be observed that the mean values of $\frac{{CoVaR}_{CSI300\left(D\right)|Other(D)}^{\beta ,t}}{{VaR}_{CSI300,D}^{\alpha ,t}}$ are greater than those of $\frac{{CoVaR}_{CSI300\left(D\right)|Other(U)}^{\beta ,t}}{{VaR}_{CSI300,D}^{\alpha ,t}}$, and the mean values of $\frac{{CoVaR}_{CSI300\left(U\right)|Other(U)}^{\beta ,t}}{{VaR}_{CSI300,U}^{\alpha ,t}}$ are greater than those of $\frac{{CoVaR}_{CSI300\left(U\right)|Other(D)}^{\beta ,t}}{{VaR}_{CSI300,U}^{\alpha ,t}}$, indicating that the positive and negative risk contagion effects are asymmetric, and the positive effect is stronger than the negative effect. Meanwhile, the mean values of $\frac{{CoVaR}_{CSI300\left(D\right)|Other(D)}^{\beta ,t}}{{VaR}_{CSI300,D}^{\alpha ,t}}$ are greater than those of $\frac{{CoVaR}_{CSI300\left(U\right)|Other(U)}^{\beta ,t}}{{VaR}_{CSI300,U}^{\alpha ,t}}$, and the mean values of $\frac{{CoVaR}_{CSI300\left(U\right)|Other(D)}^{\beta ,t}}{{VaR}_{CSI300,U}^{\alpha ,t}}$ are greater than those of $\frac{{CoVaR}_{CSI300\left(D\right)|Other(U)}^{\beta ,t}}{{VaR}_{CSI300,D}^{\alpha ,t}}$ except in CSI300-GSPTSE pairwise returns, implying the asymmetry between upside and downside risk contagion effects, and the downside effect is generally stronger, while the opposite effect is in negative contagion. The analyses are statistically supported by K-S tests (see in Table A3 and Table A4 of Appendix A).

## 6. Conclusions

The risk contagion between Chinese and mature markets has attracted more and more attention from both scholars and market participants. In this work, we construct a novel Ms-M-Clayton copula model to identify both positive and negative dependences and revisit the risk contagion between Chinese market and six mature markets in the Americas, Europe, and Asia–Oceania. Four basic Clayton copulas with various rotations are weighted to capture different tail correlations, and a two-state transition mechanism following Markov chain is introduced to allow the copula depicting dynamic risk correlations. Based on the estimated results, we calculate the CoVaR to measure the risk contagion between markets. The major conclusions are as follows:

Firstly, the financial risk dependence structures are asymmetric, and the correlations are heterogeneous. Overall, the positive dependence is dominant between Chinese and mature markets. Meanwhile, the downside risk correlation is stronger than the upside one between Chinese and American, German, and Japanese markets, while the opposite relevance is observed for Chinese and Canadian, British, and Australian markets. It is noted that compared to the Americas and European markets, the risk correlation between Chinese and Asia–Oceania markets is relatively weak. Moreover, the negative dependence should not be ignored as it may emerge in a volatile market environment and provide market participants with signals to manage their exposure. Then, the financial risk contagion is also asymmetric, which manifests in both positive and negative contagion effects, as well as in both upside and downside contagion effects. Overall, the positive effect is stronger than the negative effect, and the downside effect is stronger than the upside effect in positive structure. Compared with mature markets in Europe and the Americas, the risk spillover from Asia–Oceania markets is relatively weak, indicating that the Japanese and Australian markets can be considered as a potential choice for the investors in the Chinese market to diversify their portfolios.

This work enriches the understanding of financial risk contagion mechanism of Chinese and mature markets, which provides both practical and policy implications for investor and supervisors. With respect to practical aspects, before constructing an international portfolio, it is necessary for investors to use such quantitative models to identify and filter out markets with stronger downside risk correlation in order to better diversify their investment risks. In this study, the Chinese stock market generally has weaker risk relationship and contagion effects with mature markets in Asia–Oceania compared to the Americas and Europe thus, the Japanese and Australian markets can be regarded as feasible choices for Chinese market investors to diversify investment risks. In addition, since the Ms-M-Clayton has the capability to detect negative risk dependence structures, it is possible for investors to leverage it to discover the unusual opportunities to hedge investment risk by constructing cross-market portfolios. In the policy-making perspective, for the emerging markets at a disadvantage in risk contagion, it is essential to improve their financial system and decrease the pressure of capital outflows under extreme conditions. Specifically, according to the findings of this work, the dependence structures between markets are generally stable, which creates the possibility for supervisors to predict future risk scenarios and formulate guiding or regulatory policies using the Ms-M-Clayton copula. Moreover, as the model is sensitive to the transition probability in risk dependence states, and the supervisors are able to perceptively monitor the potential risk changes and implement risk prevention measures on previous experience. Furthermore, the Ms-M-Clayton copula model is also applicable for the series analyses of various engineering fields.

To mention, we focus only on the risk contagion between Chinese and mature stock markets in this work. Several fast-growing economies, such as Brazil, Russia, India, and South Africa, constituting the BRICS group with China, represent over 18% of the population and approximately 8% of the GDP around the world. A comparative analysis of their stock markets may be a promising topic in future research.

## Figures and Tables

**Table 1:** Descriptive statistics of monthly returns.

	CSI300	S&P500	GSPTSE	DAX30	FTSE100	Nikkei225	ASX200
Mean	0.0096	0.0062	0.0030	0.0059	0.0013	0.0046	0.0023
Max.	0.2463	0.1194	0.0997	0.1550	0.1155	0.1401	0.0949
Min.	−0.2991	−0.1856	−0.2168	−0.2131	−0.1413	−0.2722	−0.2380
Std.	0.0858	0.0436	0.0404	0.0543	0.0402	0.0570	0.0428
Skew.	−0.524	−0.888	−1.649	−0.809	−0.668	−0.896	−1.470
Kurt.	4.691	5.245	9.930	5.010	4.236	5.347	7.963
J-B.	30.685 ^a^	63.540 ^a^	456.478 ^a^	51.620 ^a^	25.654 ^a^	67.595 ^a^	257.900 ^a^
Pearson.	1.000	0.402 ^a^	0.403 ^a^	0.374 ^a^	0.307 ^a^	0.361 ^a^	0.380 ^a^

Note: superscript a represent the significant levels at 1%.

**Table 2:** M-Clayton copula estimates of CSI300 with mature stock indexes.

	CSI300-S&P500	CSI300-GSPTSE	CSI300-DAX30	CSI300-FTSE100	CSI300-Nikkei225	CSI300-ASX200
$\alpha_{1}$	0.682 ^a^	2.194 ^a^	0.889 ^a^	0.892 ^a^	0.488 ^a^	0.969 ^a^
$\alpha_{2}$	2.68 × 10^−7 a^	8.67 × 10^−8 a^	1.69 × 10^−7 a^	3.24 × 10^−8 a^	49.415 ^a^	5.23 × 10^−7 a^
$\alpha_{3}$	0.674 ^a^	0.513 ^a^	2.587 ^a^	7.08 × 10^−9 a^	0.506 ^a^	4.705 ^a^
$\alpha_{4}$	3.0967 ^a^	5.58 × 10^−8 a^	1.18 × 10^−7 a^	9.049 ^a^	5.946 ^a^	2.45 × 10^−7 a^
ω	0.924 ^a^	0.594 ^a^	0.571 ^a^	0.923 ^a^	0.947 ^a^	0.443 ^a^
Log-*L*	−13.473	−8.808	−10.369	−10.453	−10.075	−9.776

Note: superscript a represent the significant levels at 1%.

**Table 3:** MS-M-Clayton copula estimates of CSI300 with mature stock indexes.

Copula	CSI300-S&P500	CSI300-GSPTSE	CSI300-DAX30	CSI300-FTSE100	CSI300-Nikkei225	CSI300-ASX200
$\alpha_{1}^{S1}$	3.176	0.262 ^a^	0.652 ^a^	1.622	0.527 ^a^	0.243 ^a^
$\alpha_{2}^{S1}$	1.82 × 10^−10 a^	9.12 × 10^−8 a^	25.801 ^a^	4.11 × 10^−9 a^	54.908 ^a^	2.68 × 10^−8 a^
$\alpha_{3}^{S1}$	0.601 ^c^	5.594 ^a^	0.998 ^a^	3.278 ^a^	2.66 × 10^−9 a^	0.141
$\alpha_{4}^{S1}$	3.043	8.28 × 10^−8 a^	3.95 × 10^−9 a^	1.70 × 10^−9 a^	3.62 × 10^−9 a^	1.770
$\alpha_{1}^{S2}$	0.584 ^a^	1.637 ^a^	0.275	0.653 ^a^	0.461 ^a^	1.627
$\alpha_{2}^{S2}$	1.95 × 10^−10 a^	3.23 × 10^−8 a^	1.11 × 10^−10 a^	3.28 × 10^−9 a^	3.24 × 10^−10 a^	0.094
$\alpha_{3}^{S2}$	0.616 ^a^	1.39 × 10^−7 a^	20.078 ^a^	1.93 × 10^−10 a^	0.776 ^a^	5.363 ^a^
$\alpha_{4}^{S2}$	3.077	8.03 × 10^−9 a^	5.89 × 10^−10 a^	6.832 ^a^	6.844 ^a^	1.37 × 10^−8 a^
$\omega^{S1}$	0.656 ^b^	0.984 ^a^	0.978 ^a^	0.827 ^a^	0.676 ^a^	1.000 ^a^
$\omega^{S2}$	0.999 ^a^	0.676 ^a^	0.415 ^c^	0.817 ^a^	0.971 ^a^	0.932 ^c^
$P_{11}$	0.517	0.943 ^a^	0.846 ^a^	0.941 ^a^	0.943 ^a^	0.881 ^a^
$P_{22}$	0.864 ^a^	0.974 ^a^	0.710 ^a^	0.971 ^a^	0.993 ^a^	0.727 ^a^
Log-*L*	−13.626	−11.276	−12.487	−12.369	−11.522	−11.777

Note: superscript a, b, and c represent the significant levels at 1%, 5%, and 10%, respectively.

**Table 4:** Tail correlation coefficients between CSI300 and mature stock indexes.

	State 1				State 2			
	$\lambda_{LL}$	$\lambda_{LU}$	$\lambda_{UU}$	$\lambda_{UL}$	$\lambda_{LL}$	$\lambda_{LU}$	$\lambda_{UU}$	$\lambda_{UL}$
CSI300-S&P500	0.264	0.000	0.103	0.137	0.152	0.000	0.162	0.001
CSI300-GSPTSE	0.035	0.000	0.435	0.000	0.221	0.000	0.000	0.000
CSI300-DAX30	0.169	0.011	0.244	0.000	0.017	0.000	0.200	0.000
CSI300-FTSE100	0.270	0.000	0.334	0.000	0.141	0.000	0.000	0.083
CSI300-Nikkei225	0.091	0.160	0.000	0.000	0.108	0.000	0.199	0.013
CSI300-ASX200	0.029	0.000	0.004	0.000	0.304	0.000	0.410	0.000

**Table 5:** Invariant copula estimates of the CSI300 with mature stock indexes.

Copula	CSI300-SP500	CSI300-GSPTSE	CSI300-DAX30	CSI300-FTSE100	CSI300-Nikkei225	CSI300-ASX200
Gaussian						
ρ	0.372 ^a^	0.348 ^a^	0.361 ^a^	0.266 ^a^	0.314 ^a^	0.349 ^a^
Log-*L*	−12.496	−10.781	−11.660	−6.077	−8.637	−10.853
Student’s *t*						
ρ	0.372 ^a^	0.347 ^a^	0.375 ^a^	0.285 ^a^	0.314 ^a^	0.356 ^a^
v	99.899 ^a^	99.983 ^a^	7.966 ^a^	7.527 ^a^	99.320 ^a^	8.910 ^a^
Log-*L*	−12.474	−10.596	−12.438	−7.069	−8.634	−11.710
Gumbel						
δ	1.223 ^a^	1.183 ^a^	1.260 ^a^	1.141 ^a^	1.183 ^a^	1.228 ^a^
Log-*L*	−6.541	−4.251	−8.307	−2.477	−4.729	−6.603
180° rotated Gumbel						
δ	1.326 ^a^	1.281 ^a^	1.329 ^a^	1.259 ^a^	1.256 ^a^	1.316 ^a^
Log-*L*	−15.498	−11.794	−14.612	−10.533	−10.484	−14.249
Clayton						
ρ	0.630 ^a^	0.543 ^a^	0.594 ^a^	0.523 ^b^	0.505 ^a^	0.593 ^a^
Log-*L*	−16.645	−13.235	−14.419	−12.023	−12.126	−14.634
180° rotated Clayton						
ρ	0.307 ^c^	0.263	0.355 ^c^	0.130	0.237	0.310 ^c^
Log-*L*	−4.233	−3.046	−5.244	−0.671	−2.478	−4.320
SJC						
$\lambda_{U}$	2.83 × 10^−7^	4.77 × 10^−7^	5.57 × 10^−8^	1.85 × 10^−7^	4.21 × 10^−7^	4.96 × 10^−7^
$\lambda_{L}$	0.380	0.404	0.366 ^a^	0.346	0.320	0.354
Log-*L*	−16.508	−11.868	−14.474	−12.044	−11.464	−14.735

Note: superscript a, b, and c represent the significant levels at 1%, 5%, and 10%, respectively.

**Table 6:** TVP copula estimates of the CSI300 with mature stock indexes.

Copula	CSI300-S&P500	CSI300-GSPTSE	CSI300-DAX30	CSI300-FTSE100	CSI300-Nikkei225	CSI300-ASX200
TVP-Gaussian						
$\psi_{0}$	0.255 ^a^	1.162 ^a^	0.091 ^a^	0.711 ^a^	0.321 ^a^	1.601 ^a^
$\psi_{1}$	0.270 ^a^	0.258 ^a^	−0.169 ^a^	0.023 ^a^	−0.049 ^a^	−0.739 ^a^
$\psi_{2}$	1.227 ^a^	−1.423 ^a^	2.042 ^a^	−0.635 ^a^	1.117 ^a^	−1.762 ^a^
Log-*L*	−13.283	−10.836	−13.771	−6.078	−8.661	−11.657
TVP-180° Rotated Gumbel						
$\omega_{L}$	2.435 ^a^	1.144 ^a^	2.800 ^a^	1.557 ^a^	0.993 ^a^	−0.429 ^a^
$\alpha_{L}$	−0.815 ^a^	−0.311 ^a^	−0.768 ^a^	−0.557 ^a^	−0.324 ^a^	0.683 ^a^
$\beta_{L}$	−2.851 ^a^	−0.766 ^a^	−5.030 ^a^	−1.291 ^a^	−0.263 ^a^	0.340 ^a^
Log-*L*	−18.779	−11.955	−17.436	−10.738	−10.512	−14.862
TVP-Gumbel						
$\omega_{U}$	2.338 ^a^	2.903 ^a^	3.366 ^a^	3.106 ^a^	−0.654 ^a^	−0.608 ^a^
$\alpha_{U}$	−0.916 ^a^	−0.744 ^a^	−1.018 ^a^	−1.363 ^a^	0.942 ^a^	0.620 ^a^
$\beta_{U}$	−2.541 ^a^	−6.164 ^a^	−6.821 ^a^	−4.265 ^a^	−0.110 ^a^	1.100 ^a^
Log-*L*	−9.867	−9.881	−15.758	−7.732	−5.015	−8.784
TVP-SJC						
$\omega_{U}$	−14.830 ^a^	−14.363 ^a^	−15.343 ^a^	−15.242 ^a^	−14.593 ^a^	−14.490 ^a^
$\alpha_{U}$	−0.012 ^a^	−0.002 ^b^	−8.391 × 10^−4 c^	−0.002 ^a^	−0.002 ^a^	−5.799 × 10^−4 b^
$\beta_{U}$	−0.003 ^a^	7.327 × 10^−5^	4.025 × 10^−6^	−1.465 × 10^−6^	−1.469 × 10^−5^	−1.642 × 10^−4^
$\omega_{L}$	2.792 ^a^	0.459 ^a^	5.150 ^a^	4.447 ^a^	−0.181 ^a^	−2.235 ^a^
$\alpha_{L}$	−5.960 ^a^	−2.988 ^a^	−18.110 ^a^	−15.809 ^a^	−1.477 ^a^	1.071 ^a^
$\beta_{L}$	−4.505 ^a^	−1.209 ^a^	−4.230 ^a^	−4.203 ^a^	−0.858 ^a^	3.771 ^a^
Log-*L*	−19.595	−12.379	−17.450	−12.804	−11.371	−14.976

Note: superscript a, b, and c represent the significant levels at 1%, 5%, and 10%, respectively.

**Table 7:** Summary statistics of the VaR and the CoVaR (The ${CoVaR}_{CSI300\left(D\right)|Other(D)}^{\beta ,t}$ and ${CoVaR}_{CSI300\left(U\right)|Other(D)}^{\beta ,t}$ denote the downside and upside VaRs of the CSI300 conditional on the extreme declines of mature markets, respectively; the ${CoVaR}_{CSI300\left(D\right)|Other(U)}^{\beta ,t}$ and ${CoVaR}_{CSI300\left(U\right)|Other(U)}^{\beta ,t}$ denote the downside and upside VaRs of the CSI300 conditional on the extreme rises of mature markets, respectively).

	CSI300-S&P500	CSI300-GSPTSE	CSI300-DAX30	CSI300-FTSE100	CSI300-Nikkei225	CSI300-ASX200
${VaR}_{CSI300,D}^{\alpha ,t}$	−12.258 (4.483)					
${VaR}_{CSI300,U}^{\alpha ,t}$	14.720 (4.227)					
${CoVaR}_{CSI300\left(D\right)|Other(D)}^{\beta ,t}$	−19.060 (6.337)	−18.840 (6.335)	−18.791 (6.271)	−19.021 (6.228)	−18.275 (6.143)	−18.369 (6.126)
${CoVaR}_{CSI300\left(D\right)|Other(U)}^{\beta ,t}$	−7.773 (3.309)	−12.474 (5.122)	−8.736 (3.646)	−11.459 (4.664)	−10.170 (4.875)	−9.026 (3.706)
${CoVaR}_{CSI300\left(U\right)|Other(D)}^{\beta ,t}$	13.260 (3.827)	14.060 (5.324)	10.639 (3.344)	16.491 (5.574)	12.942 (5.057)	11.247 (3.468)
${CoVaR}_{CSI300\left(U\right)|Other(U)}^{\beta ,t}$	21.470 (6.056)	19.090 (4.497)	21.704 (6.116)	18.901 (4.802)	21.119 (5.988)	20.147 (5.650)

Note: this table reports the means and the standard errors (in parentheses) of VaR and CoVaR.

**Table 8:** The hypothesis testing for equalities of CoVaR and VaR.

Null Hypotheses	CSI300-S&P500	CSI300-GSPTSE	CSI300-DAX30	CSI300-FTSE100	CSI300-Nikkei225	CSI300-ASX200
${CoVaR}_{CSI300\left(D\right)|Other(D)}^{\beta ,t}={VaR}_{CSI300,D}^{\alpha ,t}$	0.593 ^a^ (0.000)	0.577 ^a^ (0.000)	0.577 ^a^ (0.000)	0.582 ^a^ (0.000)	0.550 ^a^ (0.000)	0.550 ^a^ (0.000)
${CoVaR}_{CSI300\left(D\right)|Other(U)}^{\beta ,t}={VaR}_{CSI300,D}^{\alpha ,t}$	0.582 ^a^ (0.000)	0.077 (0.637)	0.456 ^a^ (0.000)	0.159 ^b^ (0.017)	0.330 ^a^ (0.000)	0.445 ^a^ (0.000)
${CoVaR}_{CSI300\left(U\right)|Other(D)}^{\beta ,t}={VaR}_{CSI300,U}^{\alpha ,t}$	0.181 ^a^ (0.004)	0.220 ^a^ (0.000)	0.478 ^a^ (0.000)	0.132 (0.077)	0.324 ^b^ (0.047)	0.412 ^a^ (0.000)
${CoVaR}_{CSI300\left(U\right)|Other(U)}^{\beta ,t}={VaR}_{CSI300,U}^{\alpha ,t}$	0.533 ^a^ (0.000)	0.456 ^a^ (0.000)	0.544 ^a^ (0.000)	0.418 ^a^ (0.000)	0.517 ^a^ (0.000)	0.473 ^a^ (0.000)

Note: superscript a and b represent the significant levels at 1% and 5% respectively.

**Table 9:** Summary statistics of the CoVaR/VaR.

	CSI300-SP500	CSI300-GSPTSE	CSI300-DAX30	CSI300-FTSE100	CSI300-Nikkei225	CSI300-ASX200
$\frac{{CoVaR}_{CSI300\left(D\right)|Other(D)}^{\beta ,t}}{{VaR}_{CSI300,D}^{\alpha ,t}}$	1.571 (0.082)	1.551 (0.082)	1.548 (0.079)	1.569 (0.080)	1.504 (0.069)	1.513 (0.080)
$\frac{{CoVaR}_{CSI300\left(D\right)|Other(U)}^{\beta ,t}}{{VaR}_{CSI300,D}^{\alpha ,t}}$	0.625 (0.056)	1.014 (0.161)	0.707 (0.101)	0.926 (0.094)	0.828 (0.264)	0.729 (0.054)
$\frac{{CoVaR}_{CSI300\left(U\right)|Other(D)}^{\beta ,t}}{{VaR}_{CSI300,U}^{\alpha ,t}}$	0.903 (0.056)	0.943 (0.162)	0.721 (0.063)	1.110 (0.113)	0.871 (0.185)	0.762 (0.051)
$\frac{{CoVaR}_{CSI300\left(U\right)|Other(U)}^{\beta ,t}}{{VaR}_{CSI300,U}^{\alpha ,t}}$	1.461 (0.055)	1.317 (0.110)	1.478 (0.059)	1.299 (0.118)	1.441 (0.111)	1.372 (0.061)

Note: this table presents the means and the standard errors (in parentheses) of the CoVaR/VaR.

## Data Availability

The data is available on request.

## Appendix A

**Table A1:** Diagnostic tests of stationarity, autocorrelation, and heteroscedasticity.

	CSI300	S&P500	GSPTSE	DAX30	FTSE100	Nikkei225	ASX200
ADF	−11.633 ***	−12.099 ***	−11.859 ***	−12.234 ***	−13.419 ***	−11.760 ***	−12.253 ***
PP	−12.293 ***	−12.145 ***	−11.920 ***	−12.181 ***	−13.418 ***	−11.763 ***	−12.248 ***
KPSS	0.104	0.179	0.033	0.045	0.059	0.135	0.048
Q (5)	21.335 ***	6.294	7.096	7.109	3.385	4.423	3.236
Q (10)	25.902 ***	15.362	14.193	15.727	9.440	8.564	6.966
Q2(5)	28.419 ***	48.390 ***	16.423 ***	5.253	17.739 ***	5.174	11.914 **
Q2(10)	58.267 ***	52.676 ***	18.408 **	12.539	39.787 ***	10.733	14.600
ARCH (1)	0.704	26.472 ***	15.538 ***	2.126	15.553 ***	3.634 *	11.383 ***
ARCH (5)	14.973 ***	40.282 ***	17.452 ***	4.533	17.119 ***	7.610	11.441 **

Note: ***, **, and * represent the significant levels at 1%, 5%, and 10%, respectively.

**Table A2:** Parameter estimation results of the marginal distribution models and diagnostic tests.

	Parameters	CSI300	S&P500	GSPTSE	DAX30	FTSE100	Nikkei225	ASX200
Panel A.	$\phi_{0}$	1.348 ** (0.668)	1.092 *** (0.240)	0.282 *** (0.100)	0.589 (0.398)	0.447 (0.280)	0.659 (0.494)	0.760 *** (0.286)
AR-GARCH model	$\phi_{1}$	0.092 * (0.071)						
	$\phi_{4}$	0.189 *** (0.062)						
	$\alpha_{0}$	4.201 (4.241)	1.137 (0.937)	0.642 *** (0.211)		1.106 (1.058)	4.700 (3.963)	11.320 *** (2.144)
	$\alpha_{1}$	0.153 (0.116)	0.248 *** (0.102)	0.535 *** (0.103)		0.132 ** (0.062)	0.128 ** (0.053)	0.356 *** (0.130)
	$\beta_{1}$	0.797 *** (0.135)	0.723 *** (0.105)	0.342 *** (0.097)		0.809 *** (0.102)	0.730 *** (0.149)	
	GED.	1.193 *** (0.135)	1.286 *** (0.213)			1.652 *** (0.262)		1.494 *** (0.242)
Panel B.	Log-*L*	−627.552	−510.743	−343.105	−578.161	−511.389	−579.028	−517.528
Diagnostic tests	AIC	6.973	5.546	3.732	6.228	5.553	6.314	5.608
	Q (5)	3.820	1.698	3.799	7.109	0.712	0.474	1.594
	Q (10)	6.385	6.374	7.398	15.727	4.119	3.896	5.102
	Q2 (5)	2.549	3.639	3.505	5.971	5.056	2.588	1.393
	Q2 (10)	9.202	9.977	9.374	12.817	15.250	7.690	9.668
	ARCH (1)	0.278	1.998	2.009	2.124	2.289	0.796	0.590
	ARCH (5)	2.491	3.607	3.678	4.533	4.583	3.340	1.273

Note: ***, **, and * represent the significant levels at 1%, 5%, and 10%, respectively.

**Table A3:** The K-S test for CoVaR/VaR between positive and negative risk spillovers.

Hypotheses	CSI300-S&P500	CSI300-GSPTSE	CSI300-DAX30	CSI300-FTSE100	CSI300-Nikkei225	CSI300-ASX200
$H_{0}:\frac{{CoVaR}_{CSI300\left(D\right)|Other(D)}^{\beta ,t}}{{VaR}_{CSI300,D}^{\alpha ,t}}=\frac{{CoVaR}_{CSI300\left(D\right)|Other(U)}^{\beta ,t}}{{VaR}_{CSI300,D}^{\alpha ,t}}$; $H_{1}:\frac{{CoVaR}_{CSI300\left(D\right)|Other(D)}^{\beta ,t}}{{VaR}_{CSI300,D}^{\alpha ,t}}\neq \frac{{CoVaR}_{CSI300\left(D\right)|Other(U)}^{\beta ,t}}{{VaR}_{CSI300,D}^{\alpha ,t}}$.	1.000 *** (0.000)	1.000 *** (0.000)	1.000 *** (0.000)	1.000 *** (0.000)	0.945 *** (0.000)	0.550 *** (0.000)
$H_{0}:\frac{{CoVaR}_{CSI300\left(U\right)|Other(U)}^{\beta ,t}}{{VaR}_{CSI300,U}^{\alpha ,t}}=\frac{{CoVaR}_{CSI300\left(U\right)|Other(D)}^{\beta ,t}}{{VaR}_{CSI300,U}^{\alpha ,t}}$; $H_{1}:\frac{{CoVaR}_{CSI300\left(U\right)|Other(U)}^{\beta ,t}}{{VaR}_{CSI300,U}^{\alpha ,t}}\neq \frac{{CoVaR}_{CSI300\left(U\right)|Other(D)}^{\beta ,t}}{{VaR}_{CSI300,U}^{\alpha ,t}}$.	1.000 *** (0.000)	0.824 *** (0.000)	1.000 *** (0.000)	0.577 *** (0.000)	0.896 *** (0.000)	0.445 *** (0.000)

Note: this table summarize the results of the Kolmogorov–Smirnov (KS) tests; *** represent the significant levels at 1%, and the *p*-values for the KS statistics are reported in parentheses.

**Table A4:** The K-S test for CoVaRs/VaRs between upside and downside risk spillovers.

Hypotheses	CSI300-S&P500	CSI300-GSPTSE	CSI300-DAX30	CSI300-FTSE100	CSI300-Nikkei225	CSI300-ASX200
$H_{0}:\frac{{CoVaR}_{CSI300\left(D\right)|Other(D)}^{\beta ,t}}{{VaR}_{CSI300,D}^{\alpha ,t}}=\frac{{CoVaR}_{CSI300\left(U\right)|Other(U)}^{\beta ,t}}{{VaR}_{CSI300,U}^{\alpha ,t}}$; $H_{1}:\frac{{CoVaR}_{CSI300\left(D\right)|Other(D)}^{\beta ,t}}{{VaR}_{CSI300,D}^{\alpha ,t}}\neq \frac{{CoVaR}_{CSI300\left(U\right)|Other(U)}^{\beta ,t}}{{VaR}_{CSI300,U}^{\alpha ,t}}$.	0.615 *** (0.000)	0.797 *** (0.000)	0.440 *** (0.000)	0.830 *** (0.000)	0.247 *** (0.000)	0.412 *** (0.000)
$H_{0}:\frac{{CoVaR}_{CSI300\left(D\right)|Other(U)}^{\beta ,t}}{{VaR}_{CSI300,D}^{\alpha ,t}}=\frac{{CoVaR}_{CSI300\left(U\right)|Other(D)}^{\beta ,t}}{{VaR}_{CSI300,U}^{\alpha ,t}}$; $H_{1}:\frac{{CoVaR}_{CSI300\left(D\right)|Other(U)}^{\beta ,t}}{{VaR}_{CSI300,D}^{\alpha ,t}}\neq \frac{{CoVaR}_{CSI300\left(U\right)|Other(D)}^{\beta ,t}}{{VaR}_{CSI300,U}^{\alpha ,t}}$.	0.989 *** (0.000)	0.346 *** (0.000)	0.253 (0.217)	0.703 *** (0.000)	0.484 *** (0.000)	0.473 *** (0.000)

Note: this table summarize the results of the Kolmogorov–Smirnov (KS) tests; *** represent the significant levels at 1%, and the *p*-values for the KS statistics are reported in parentheses.
